# Oil from Mullet Roe Byproducts: Effect of Oil Extraction Method on Human Erythrocytes and Platelets

**DOI:** 10.3390/foods13010079

**Published:** 2023-12-25

**Authors:** Ioannis Tsamesidis, Paraskevi Tzika, Despoina Georgiou, Aggelos Charisis, Sakshi Hans, Ronan Lordan, Ioannis Zabetakis, Eleni P. Kalogianni

**Affiliations:** 1 Department of Food Science and Technology, Sindos Campus, International Hellenic University, 57400 Thessaloniki, Greece; johntsame@gmail.com (I.T.); tzikap@food.ihu.gr (P.T.); dgeorgiou@ihu.gr (D.G.); agcharisis@food.ihu.gr (A.C.); 2 Department of Biological Sciences, University of Limerick, V94 T9PX Limerick, Ireland; sakshi.hans@ul.ie (S.H.); ronan.lordan@pennmedicine.upenn.edu (R.L.); funfood16@gmail.com (I.Z.); 3 Institute for Translational Medicine and Therapeutics, Perelman School of Medicine, University of Pennsylvania, Philadelphia, PA 19104, USA; 4 Department of Medicine, Perelman School of Medicine, University of Pennsylvania, Philadelphia, PA 19104, USA; 5 Bernal Institute, University of Limerick, V94 T9PX Limerick, Ireland; 6 Health Research Institute, University of Limerick, V94 T9PX Limerick, Ireland

**Keywords:** hemocompatibility, mullet roe, cytotoxicity, marine oil, reactive oxygen species, oil extraction, antiplatelet therapeutics

## Abstract

**Background**: The valorization of byproducts to obtain high nutritional value foods is of utmost importance for our planet where the population is booming. Among these products are oils rich in ω-3 fatty acids produced from fishery byproducts. Recently, mullet roe oil from roe byproducts was produced that was rich in the ω-3 fatty acids eicosatetraenoic acid (EPA) and docosahexaenoic acid (DHA). Oils are customarily characterized for their composition and degree of oxidation but little is known of their biological effects, especially the effect of the extraction method. **Methods**: The purpose of this study was to evaluate the effects of freshly extracted mullet roe oil from mullet roe byproducts and the effect of the extraction method on human red blood cells (hRBCs) and platelets. To this end, the hemocompatibility (cytotoxicity), oxidative effects, and erythrocyte membrane changes were examined after 1 and 24 h of incubation. Antiplatelet effects were also assessed in vitro. **Results**: The expeller press oil extraction method and alcalase-assisted extraction produced the most biocompatible oils, as shown by hemocompatibility measurements and the absence of erythrocyte membrane alterations. Solvent extracts and protease-assisted extraction oils resulted in the rupture of red blood cells at different examined dilutions, creating hemolysis. **Conclusions**: It seems that the proper functioning of oil–erythrocyte interactions cannot be explained solely by ROS. Further investigations combining chemical analysis with oil–cell interactions could be used as an input to design high nutritional value oils using green extraction technologies. All samples exhibited promising antiplatelet and antiblood clotting effects in vitro.

## 1. Introduction

The valorization of agri-food waste has gained significant attention due to natural resource management and environmental sustainability on a worldwide scale [1]. The idea of using agri-food waste as a raw material for nutrient-dense foods, food additives, or supplements for human consumption or animal feed is gaining a lot of interest. Large quantities of waste and byproduct materials from the industrial processing of fish can be used to make protein concentrates, bioactive peptides, oils, and enzymes as well as other products with an additional value [2]. Bioactive fish molecules (proteins, lipids, and minerals) are currently used in a variety of products by a number of relevant sectors [3].

Greece produces the highly prized gourmet food known as avgotaracho and similar goods are also produced in Japan (karasumi) and Italy (bottarga) [4]. Τhe particularity of “avgotaracho Messolonghiou” from Greece has been identified by the EU (1 July 1996) as a protected designation of origin (PDO) product (EC 1263/96) [5,6]. Greek avgotaracho has expanded its reputation as a gourmet product during the past few decades and it is produced from the whole ovarian sacs of striped mullet roe. Avgotaracho production first involves scrubbing the ovarian sacs and then leaving them in a salt solution for two to three hours. They are then finalized by being placed in casts and are allowed to air-dry for three and a half days in a controlled environment. Finally, they are covered with seven layers of wax [5,6]. Not all sizes of skeins are adequate for avgotaracho or the production of similar delicacies and those not of the appropriate size are discarded. In addition, during the steps of production, some of the ovarian sacs break and cannot be used further. Overall, during the production process, approximately 10% of the ovarian sacs and their content becomes waste. Nevertheless, due to the mild conditions used during the handling of the product, the above byproducts retain the nutritional value of the initial products. According to chemical analysis, high nutritional value oils can be produced using byproducts from the manufacturing of avgotaracho [6,7]. Although avgotaracho and its associated products are frequently consumed, nothing is known about their toxicity or that of their oil extracts. Regarding the methods of oil extraction, traditional techniques for fish-oil extraction, which mainly involve wet rendering or solvent extraction, can involve heating and/or the use of organic solvents [6,8]. The former causes heat-labile compounds to oxidize and degrade, whereas the latter is hazardous to human health and the environment. Energy-saving and risk-free green extraction techniques for fish oils and protein isolates have been researched in the context of a more sustainable production [9]. These techniques include pH-shift processing, enzymatic hydrolysis, subcritical water extraction, fermentation, supercritical fluid extraction with carbon dioxide (SFE–CO_2_), ultrasound extraction, and microwave extraction [10]. Kalogianni et al. and Georgiou et al. applied physical methods as well as organic solvent extraction in order to produce food-grade mullet roe oil from avgotaracho production waste [8,9]. An expeller press and ethanol extraction methods were selected as the methods, providing good recovery and oil-quality characteristics. Another method used for the extraction of oils from fishery waste is enzymatic extraction [11,12,13,14].

Biological tests are considered to be safe alternative assays that mimic cell interactions in vitro when examining the biological properties of a treatment due to their “natural” status [15]. Hemocompatibility evaluates the compatibility of plain materials or loaded materials with drugs, proteins, oils, etc., with blood [15,16]. In detail, hemolysis determines the degree of red cell lysis and the release of hemoglobin [15,16,17] caused by materials or their extracts in vitro. Moreover, reactive oxygen species (ROS) are frequently produced by aquatic organisms in reaction to stressful factors [18], which has an impact on the quality of the end bio-product. In order to determine the antioxidant capacity of fish oil in cells, particularly in red blood cells (RBCs), it is important to assess the parameters associated with oxidative stress in avgotaracho oil, especially those that initially demonstrate a redox potential. Therefore, RBCs exposed to oxidants produced by the immune system and endothelial cells or by other external factors such as fish oils can produce or counteract superoxide, H_2_O_2_, hypochlorous acid (HOCl), nitric oxide (NO•), and peroxynitrite (ONOO−) radicals. As a result, the cytosolic RBC antioxidant system can largely be affected or empowered by the external factors ameliorating the redox imbalance.

In this paper, we first focus on the hemocompatibility (cytotoxicity), the oxidative effects, and the erythrocyte membrane alterations of freshly extracted avgotaracho oil on human red blood cells (hRBCs) following the use of various extraction methods. To the best of the authors’ knowledge, there is no previous study that examines the biological effects of mullet roe oil or mullet roe oil byproducts. For the extraction, a physical extraction method and a solvent extraction method—namely, expeller press extraction and ethanol extraction—were selected according to our previous work as methods that could provide a good extraction yield and good oil-quality characteristics as determined by chemical analysis [8,9]. In addition, enzymatic extraction, which is often used for the production of oils from fish byproducts [11,12], was used for comparison. Alcalase and protease were chosen. Taking into consideration that the digestive tube receives a prodigious blood supply, mainly from the branches of the aorta and the digestion and absorption process of oils in general, it is of great interest to set the toxicological profile of new oils before they enter the market [15]. There is also significant evidence in the literature of the health benefits of marine lipids with regards to their anti-inflammatory and antiplatelet properties. Platelet agonists such as thrombin and platelet-activating factor (PAF) are important mediators of platelet activation and coagulatory pathways that act via their G-protein-coupled receptors [19]. Thrombin is a protease molecule that plays an important role in platelet activation, blood clot formation, and the coagulatory cascade. The overactivation of the thrombin pathway is associated with atherosclerosis and cardiovascular events [19]. In drug development, various molecules have been found to be able to suppress the process of blood clotting and, consequently, platelet aggregation through different pathways. These compounds can be utilized to treat and prevent cardiovascular disease (CVD), which is linked to blood platelet hyperactivation [19]. In our study, we investigated the effect of mullet roe lipids on human platelet activity against the thrombin pathway of platelet activation and the blood clotting time in vitro. Moreover, considering that the enzymes that are involved in energy production and redox reactions largely trigger oxidative damage to RBCs [20] and in order to understand their potential antioxidant properties and potential protection from oxidative injury [21] as previously reported with other substances (anthocyanins) [22], we investigated their oxidative stress levels in red blood cells.

## 2. Materials and Methods

### 2.1. Flow Diagram of the Study

**Figure 1:**
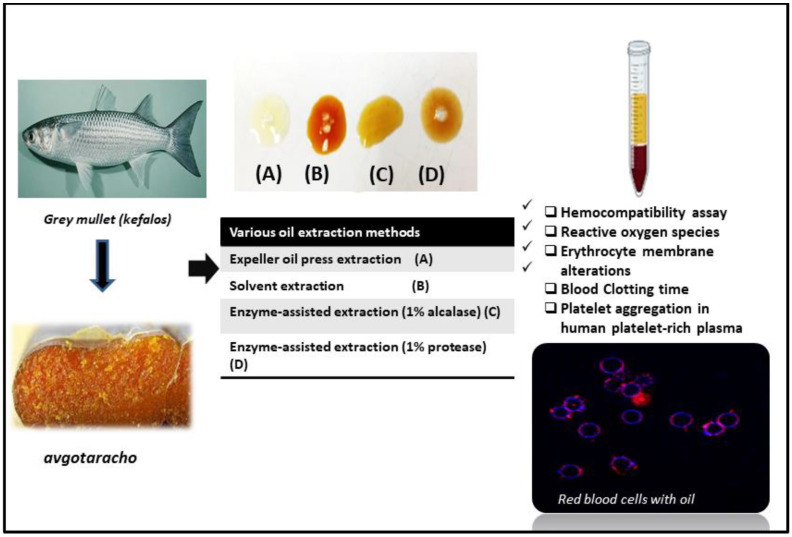
Graphical representation of the experimentation.

Figure 1 presents the flow diagram of the present study.

### 2.2. First Materials and Chemicals

Mullet roe byproducts were donated by Tricalinos Co. (Athens, Greece). Food-grade ethanol (95%) was purchased from Alcovin S.A. (Athens, Greece). Alcalase^®^ enzymes from *Bacillus licheniformis* were purchased from Sigma-Aldrich Chemie GmbH (Darmstadt, Germany) (activity ≥ acti Anson units/mL) and protease (Protamex™) from *Bacillus* sp. was purchased from Novo Nordisk (Bagsvaerd, Denmark) (activity ≥ An U/g). P-anisidine (99%) and isooctane (spectrophotometric grade) were purchased from Sigma Aldrich (Darmstadt, Germany) and acetic acid (glacial, 99–100%) was purchased from Chem-Lab NV (Zedelgem, Belgium). Starch (soluble for analysis) and sodium thiosulfate (0.1 N) were purchased from Merck KGaA (Darmstadt, Germany) and potassium iodide (99%) was purchased from Carlo Erba Reagenti SPA (Strada Rivatana, Rodano). For the biological evaluation, the reagents used were DMSO (90%) and hydrogen peroxide (H_2_O_2_) (37%), which was purchased from Sigma Aldrich (Darmstadt, Germany). CM-H2DCFDA was purchased from MoBiTec (Goettingen, Germany). Platelet aggregation-testing reagents, including bovine serum album (BSA), dimethylsulfoxide (DMSO, 90%), and standard thrombin receptor-activating peptide 6 (TRAP-6), were purchased from Merck (Dublin, Ireland). Phlebotomy consumables, including needles (22 G) and 8.2 mL sodium citrate S-Monovettes, were purchased from Sarstedt Ltd. (Wexford, Ireland). Consumables for platelet aggregometry were purchased from Labmedics LLP (Dublin, Ireland)

### 2.3. Extraction Methodology

Table 1 presents the mullet roe oils tested and the extraction method. Details of the employed extraction are described below.

For the solvent extraction at 25 °C, 20 g of the freeze-dried and crushed sample was mixed with 200 mL ethanol and magnetically stirred for 2 h at 25 °C. After filtering, the extraction was repeated using 200 mL of ethanol for 20 min. Both extracts were mixed and centrifuged at 1878× *g* for 15 min (Rotofix 32A; Hettich, Geldermalsen, Netherlands ) and then the solvent was evaporated in a rotary vacuum evaporator (R-210 Rotavapor; Buchi, Avantor, Basel, Switzerland) at 37 °C.

For the expeller oil press extraction, 300 g of defrosted raw mullet roe was added in to an oil press (OW 500 s-inox oil press; Oelwerk, Germany) working at 50 °C. The resulting cake was added into the oil press for a second time for residual oil extraction. A nozzle with a diameter of 16 mm was used and the machine was run at 20 Hz in both cases. Following the pressing, the resulting cake, water, some solids, and extracted oil were centrifuged (RC3; Sorvall, Thermo Scientific, Dublin, Ireland) at 4132× *g* for 15 min and the fish oil was separated.

For the enzymatic extraction, 300 g of defrosted raw mullet roe was heated at 90 °C for 10 min in order to inactivate endogenous enzymes. After that, the enzyme (1% alcalase or 1% protease; i.e. 1 mL of enzyme solution/100 g of byproduct) was added at 55 °C and incubated for 35 min with continuous stirring. Finally, the added enzymes were inactivated at 90 °C for 10 min, followed by cooling at 25 °C. The extracted fish oil was separated by centrifuging the cooled mullet roe solution at 8000 rpm for 15 min at 25 °C (Sorvall; Evolution RC, Thermo Scientific, Dublin, Ireland).

### 2.4. Oil Solubilization for Biological Assays

The oil solubilization was performed using DMSO with a final concentration of approximately 0.001%. In detail, 40 μL of the tested oil was solubilized with 4 μL DMSO in 920 μL phosphate buffer saline (PBS) (initial solubilized stock). D1 was prepared using 20 μL of the solubilized oil (initial solubilized stock) in 980 μL PBS. D2 was prepared using 40 μL of the solubilized oil (initial solubilized stock) in 960 μL PBS. D3 was prepared using 80 μL of the solubilized oil (initial solubilized stock) in 920 μL PBS. The hemocompatibility assay was performed using 20, 25, and 30 μL of solubilized oil in 980, 975, and 970 erythrocyte suspensions (2% hematocrit).

### 2.5. Analysis of Oxidation Oil Levels

Fish-oil oxidation was determined using the peroxide value (PV) following the iodometric method. The K_232_ and K_268_ values were determined using isooctane (spectroscopy grade; Sigma) as the solvent by measuring the absorption at 232 nm and 268 nm, respectively, according to the European Commission Regulation (EEC) No 2568/91 (Commission Regulation (EEC) No 2568/91) [23]. The p-anisidine value (AV) was determined according to the AOCS Official Method Cd 18–90. The total oxidation (TOTOX) value was calculated as TOTOX = 2PV + AV [24].

### 2.6. Hemocompatibility and Platelet Aggregation Assay

#### 2.6.1. Blood Sample Collection

For the hemocompatibility assay, red blood cells were separated from whole blood collected from the department of blood donation at the General Hospital of Naousa, Greece. The confidentiality of the blood donors was wholly preserved. Good clinical practice guidelines and the Declaration of Helsinki were followed according to the Ethical Committee of the hospital’s approval of the study (ID_233205920). For both hemocompatibility and the platelet aggregation assay, healthy human volunteers (2 female and 1 male) (*n* = 3) donated blood samples after written consent was obtained with the knowledge that their blood samples would be used in the research project concerned. Blood samples were drawn via the venipuncture of the median cubital vein using a 22 G needle into evacuated S-Monovettes containing a sodium citrate anticoagulant for further processing and use in platelet assays. Blood collection was performed after obtaining ethical approval from the university’s ethical committee (ethical approval code: 2022_01_01_S&E) and with the written consent of all donors involved.

#### 2.6.2. Hemocompatibility (Hemolysis Assay)

Hemolysis assays with the tested oil preparations were determined using RBCs. Packed erythrocytes were then resuspended in the isotonic solution at different hematocrit values calculated using a hematocrit reader and read as % hematocrit as previously described [25]. RBCs were then prepared using a 2% volume erythrocyte (final volume: 1 mL) (hematocrit: 2%). Diluted RBCs were incubated with different concentrations of preparations as described above (oil solubilization for 24 h of incubation at 37 °C). The supernatant of the untreated RBCs was used as the negative control (Ctrl-) and RBCs treated with distilled water were used as the positive control. The absorbance value of hemoglobin at 541 nm was measured with the reference wavelength of 700 nm. The percent of hemolysis was calculated as follows:

$$\mathrm{Hemolysis}\;\%\;=\;\frac{\mathrm{sample}\;\mathrm{absorbance}\;-\;\mathrm{negative}\;\mathrm{control}}{\mathrm{positive}\;\mathrm{control}\;-\;\mathrm{negative}\;\mathrm{control}}\;\times \;100\%$$

The experimental procedure was performed in quintuplicate (*n* = 5).

### 2.7. Fluorescence Analysis for the Detection of ROS Levels

For the detection of intracellular reactive oxygen species (ROS), a cell-permeable probe (2′,7′-dichlorodihydrofluorescein diacetate, Merck, St. Louis, MO, USA) was used. ROS baseline levels were detected after the incubation of hRBCs with the tested oils. The oxidation of CM-H2DCFDA (chloromethyl derivative of H2DCFDA) (prepared as a 0.5 mM stock solution in DMSO) in RBCs was monitored by the measurement of the fluorescence of the desired RBC suspension (0.2% Hematocrit) in 96-well black-walled microplates (Corning^®^; catalogue number 3340, Life Sciences, Flintshire, UK) using a Shimadzu fluorescence spectrophotometer. The relative fluorescence was expressed as “% maximal emission”, where maximal emission (control+) was defined as the fluorescence emission obtained following the addition of 3 mM H_2_O_2_ as a negative control for the RBC solution without the oil treatment.

### 2.8. Confocal Laser Scanning Microscopy (CLSM)

The same set of experiments used for the hemocompatibility assays was repeated for the imaging of the cells after the interactions with different oil types. To stain the erythrocyte membrane, cells were acquired from fresh blood, washed, and incubated with the 4 oil types as previously described (see Hemocompatibility section). A small amount of the 10 μL suspension was incubated for 10 min with Nile Red and Nile Blue. The slide was then held under a microscope. Microstructure images of erythrocytes were acquired using a CLSM, model EVO 50XVP (Carl Zeiss, CZ Miscoscopy GmbHm, Jena, Germany). The Ar/K and He/Ne dual-channel laser mode was used. Nile Red and Nile Blue were excited with a laser at wavelengths of 625 nm and 660, respectively. Image acquisition was performed using a 40× and 60× oil lens.

### 2.9. Analysis of Platelet Aggregation in Human Platelet-Rich Plasma (hPRP) of Fish Oils and Blood Clotting Time

#### 2.9.1. Ethics Statement for Platelets

Venous blood was drawn from 3 healthy individuals (2 female and 1 male). All subjects provided written informed consent before entering the study. Ethical approval for the platelet aggregation studies was obtained from the Ethics Committee of the University of Limerick (ethical approval number: 2022_01_01_S&E). The study was approved by the local Ethics Committee and conducted in accordance with good clinical practice guidelines and the Declaration of Helsinki. The donor exclusion criteria were use of medication for cardiovascular disorders, taking anti-inflammatory drugs, not smoking, or health issues such as a heart condition or blood clotting disorder.

In detail, blood samples collected in S-Monovettes (as described in Section 2.6) were centrifuged at 194× *g* for 18 min at 20 °C with no brake applied using an Eppendorf 5702R centrifuge (Eppendorf Ltd.; Stevenage, UK). The resulting supernatant (PRP) was then transferred to polypropylene tubes at room temperature for use in the platelet aggregation assays and platelet-poor plasma (PPP) was obtained by further centrifuging the specimens at 1465× *g* for 20 min at 20 °C with no brake applied. The platelet concentration of the PRP was adjusted to 500,000 platelets/µL if required by dilution with PPP according to the absorbance of PRP measured using a Shimadzu UV-1800 spectrophotometer (Kyoto, Japan) before analysis using a Chronolog-490 two-channel platelet aggregometer.

Standard TRAP-6, obtained from Merck, was prepared in distilled sterile H_2_O at a working concentration of 0.5 mM. Fish-oil dilutions were prepared as follows. An initial solubilized stock of fish oil in DMSO was prepared at a ratio of 1:2 to fully dissolve the oils. From this, a dilution (solution B) was prepared in 1X PBS so that the concentration of DMSO was 0.1%. Another serial dilution (solution A) was prepared from this so that the concentration of DMSO was 0.01%. This low concentration was maintained in order to minimize the effect of DMSO on platelet inhibition. Solution A was used as the final working solution in the platelet analysis.

The analysis of platelet aggregation was carried out using a Chronolog-490 two-channel turbidimetric platelet aggregometer (Havertown, PA, USA) coupled to the accompanying AGGRO/LINK 8 software package. At the start of the procedure, 250 µL PRP was added to an aggregometer cuvette at 37 °C and stirred at 1200 rpm. The PRP was calibrated using the PPP as a blank. A certain volume of TRAP-6 (0.5 mM) was added to the cuvettes in order to induce maximum reversible aggregation in the absence of any lipid samples. For each lipid sample, the mass of lipid required to inhibit 50% of the TRAP-6-induced aggregation was calculated. From this, the IC_50_ was calculated as previously described [26].

#### 2.9.2. Assessment of Blood Clotting Time (BCT)

The BCT was estimated as previously described [27]. The same fish dilutions as for the analysis of platelet aggregation in hPRP were used. Each sample was placed in Eppendorf tubes in a water bath at 37 °C for 10 min. After adding 20 μL of 0.2 M CaCl_2_ to the tubes with whole blood (340 μL), they were heated in a water bath at 37 °C. The time that no flow was seen following tube inversion (the tubes were inverted every 10 s and remained in place for 1 s before reverting) was called the BCT. Without any materials, the control was measured. Three observers counted all the tested samples.

All experiments were performed in triplicate (*n* = 3) and the resulting IC_50_ values were expressed as the mean value of the mass of lipid (µg) in the aggregometer cuvette ± standard deviation (SD).

### 2.10. Statistical Analysis

The oil oxidation parameters were statistically assessed using an analysis of variance (ANOVA) at *p* = 0.05, then the averages were compared using Tukey’s test. Minitab 21.4.2 (Minitab Inc., State College, PA, USA) statistical software was used for this. To compare the different extraction methods for hemocompatibility, the ROS, and the blood clotting experiments, the statistical analysis was performed using SPSS software, version 22.0. Descriptive statistics, presented as the mean ± standard deviation, were obtained. Additionally, an inferential statistical analysis (*t*-test) was used to investigate the possible differences between the different extraction methods. In all the statistical analyses, the level of significance (*p*-value) was set at α = 0.05.

## 3. Results and Discussion

### 3.1. Oil Oxidation

Table 2 presents the oxidation of the samples, including the primary and secondary oxidation products (namely, the peroxide value (PV), the K_232_ and K_268_ indices, the anisidine value (AV), and TOTOX value) of the oils extracted by the different extraction processes. Regarding the formation of primary oxidation products, all oils presented a low PV and conjugated dienes (K_232_) formation, even without the use of antioxidants during the oil extraction. The PV was below the limits set by the codex alimentation standards (CAC) for human consumption (PV < 5 meq O_2_/kg oil) [28]. Regarding the secondary oxidation products, the AV values remained within the limits set by CAC (AV < 20) and both the AV and K_268_ were strongly affected by the applied method. Overall, enzymatic extraction resulted in much higher concentrations of secondary oxidation products and the expeller press provided the best results in terms of all oxidation indices. The effect of non-enzymatic processes on mullet roe oil quality has recently been examined in detail by Kalogianni et al. [9] and Georgiou et al. [8], highlighting that process factors have an impact on oil quality in terms of oxidation.

### 3.2. Hemocompatibility and Erythrocyte Membrane Alterations of Mullet Roe Oil Extracts

**Figure 2:**
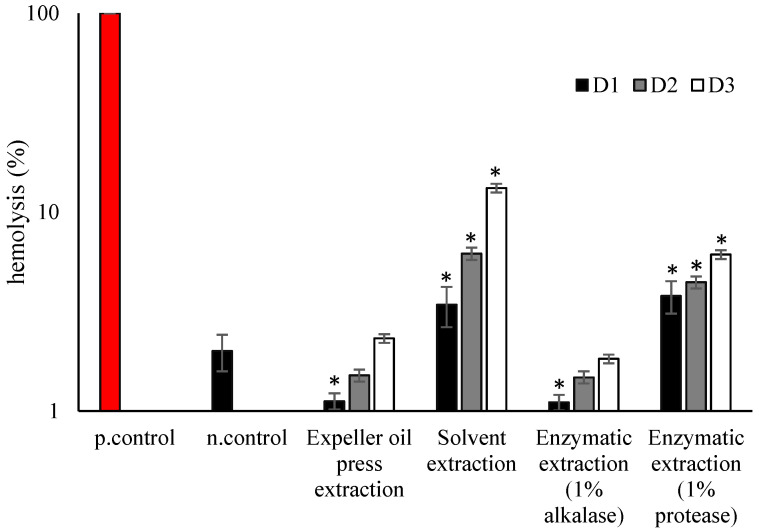
Hemocompatibility of mullet roe oil after incubation with healthy erythrocytes at 37 °C. p.control denotes the positive control and n.control denotes the negative control. * indicates statistically significant difference (*p* < 0.05) between n.control and oil-treated erythrocytes (D1: dilution 1; D2: dilution 2; D3: dilution 3) (see Section 2.4 for abbreviations).

All the examined extracts did not present hemolysis under all the tested concentrations after 1 h of incubation. This phenomenon indicated that the reaction of erythrocytes with oil extracts was not direct, but needed some time for initiation. For this reason, the hemolysis rates were also measured 24 h after the incubation (Figure 2).

It was found that mullet roe oil extracts using different methods affected the hemocompatibility of human red blood cells (hRBCs). Specifically, mullet roe oil after expeller oil press extraction presented no hemolytic effect (1–2%) under all tested dilutions. On the other hand, the oil from solvent extraction presented higher hemolytic effects (3 to 13% hemolysis) for all examined dilutions in comparison with the expeller method. Regarding the enzymatic extracts, alcalase did not affect the red cell membrane. However, the enzymatic extraction with protease created hemolysis even at low oil dosages. The enzymatic approach with alcalase as the enzyme of choice appeared to be more compatible compared with protease. We have demonstrated in this research study that erythrocytes, after incubation with solvent-extracted oil at all tested dosages, initiated a hemolytic crisis. The hemolysis of the oils was not solely related to the level of oil oxidation, and the biological methods provided further information on the effects of the oils on human cells. Among the methods used, the highest hemocompatibility was observed for the mullet byproduct oil produced using the expeller press; the lowest was by solvent extraction. One of the oldest and most basic techniques to obtain oil is an expeller press, often known as an oil press. The mechanical crushing technique, which is similar in principle, is also utilized to extract oil from algal biomass and seeds in a straightforward, yet efficient, manner [29]. Moreover, it has been used to optimize the design of presses for fish-oil extraction [30]. Among the enzymatic extraction methods, the method that had the lowest impact on oil oxidation (the method using alcalase) also had the best results in terms of hemocompatibility.

**Figure 3:**
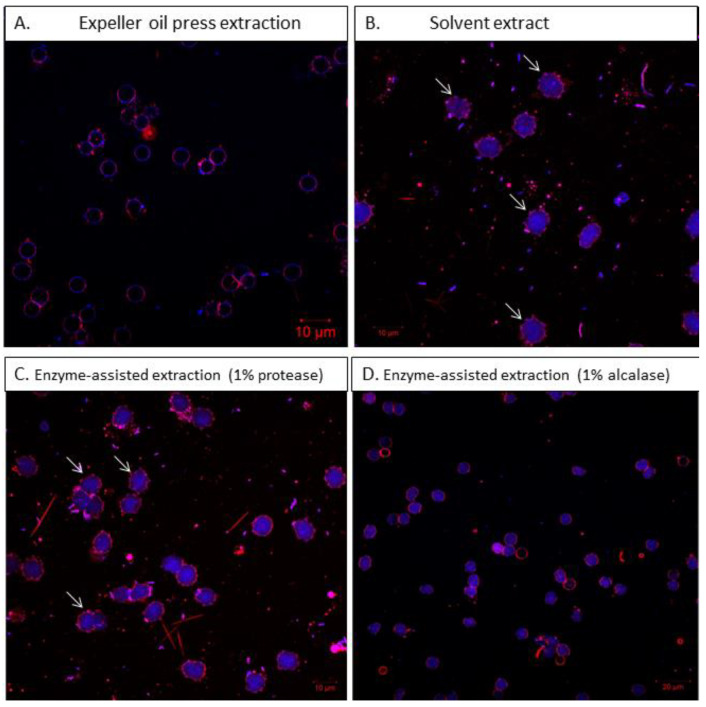
CLSM images of erythrocytes treated with mullet roe oil from different extraction methods. The white arrows indicate the erythrocyte membrane damage (10 μm scale bar). Images (**A**–**D**) correspond with oils from different processes. All measurements were performed at 37 °C.

To better understand the biocompatibility of the extracted oil, the erythrocyte membrane alterations were monitored with the use of confocal microscopy. Figure 3 presents the erythrocyte membrane modifications after incubation with different oil extracts. The microscope observation showed changes in the shape and color intensity of red blood cells at the highest oil concentration tested of the solvent oil extract (Figure 3B) and protease-assisted extraction (Figure 3C). Erythrocytes incubated with the expeller oil (Figure 3A) and alcalase-assisted extraction (Figure 3D) did not change in shape, retaining their oval architecture. At concentrations higher than 80 μL of solubilized oil, red blood cells showed mild hypochromia as well as changes in shape and erythrocyte membrane formation. The white arrows in Figure 3 highlight the membrane modifications and transformation to acanthocytes. Thus, in addition to the elements that affect red blood cells’ plasticity (such as shape, size, cell viscosity, and membrane rigidity), the remarkable changes in the erythrocyte cytoskeletal architecture and membrane stiffness could be attributed to oxidative damage. For this reason, the ROS levels of the oil-treated erythrocytes were also investigated.

### 3.3. Reactive Oxygen Species Levels of Erythrocytes after Mullet Roe Byproduct Oil Incubation

**Figure 4:**
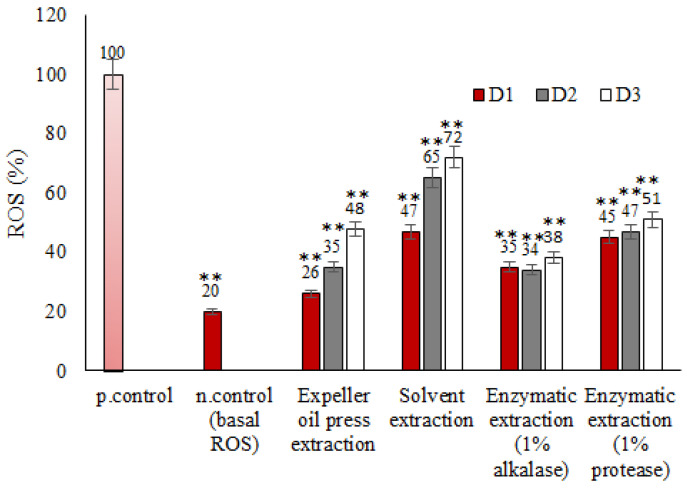
ROS levels (%) of avgotaracho oil after incubation with healthy erythrocytes at 37 °C. p.control denotes the positive control and n.control denotes the negative control. ** indicates statistically significant difference (*p* < 0.01) between n.control and oil-treated erythrocytes.

The detection of intracellular reactive oxygen species (ROS), including peroxides, superoxides, hydroxyl radicals, singlet oxygen, and alpha-oxygen, was determined using the cell-permeable probe 2′,7′-dichlorodihydrofluorescein diacetate. ROS baseline levels were detected after the incubation of hRBCs with the tested oils (Figure 4). Mullet roe oil after expeller oil press extraction presented average ROS levels ranging from 26 to 48%; on the other hand, mullet roe byproduct oil from solvent extraction ranged between 47 and 72%. The ROS levels of erythrocytes treated with oils extracted with enzymes (alcalase and protease) presented lower ROS amounts in comparison with the first two approaches, especially at high oil concentrations. Taking into consideration that eryptosis, which includes possible defensive erythrocyte mechanisms, is one of the signaling cascades that are mediated by the oxidative environment and when erythrocytes are fragile, it is possible that high concentrations of the tested fish oils triggered eryptosis and the dose-dependent ROS amounts.

The alcalase approach revealed ROS amounts between 34 and 38% and protease-assisted extraction ranged from 45 to 51%. The hemocompatibility results were in accordance with the elevated amounts of ROS levels, clarifying the central role of ROS in the hemolytic activity of the oil extracts when the latter were produced via enzymatic extraction. Alcalase-assisted extraction presented the best hemocompatibility at high oil concentrations without erythrocyte membrane alterations explained by the low ROS amounts. In detail, to better understand the role of ROS generation by cells, it should be noted that ROS have the ability to change signaling proteins and the equilibrium of redox in the blood [31,32,33]. Hemoglobin (Hb), which makes up 95–97% of RBC’s cytosolic proteins, is the primary component involved in this pathway [34,35]. The majority of other proteins aid in defending the blood cells from oxidative stress, and include superoxide dismutase (SOD), catalase (CAT), glutathione peroxidase (GPx), and peroxiredoxin 2 (PRDX2) [36]. Therefore, the main cause of oxidative stress in the erythrocyte is hemoglobin. Exogenous oxidants can also harm RBCs. The process of Hb autoxidation yields a superoxide radical, from which hydrogen peroxide is produced through catalyzed or spontaneous dismutation [37]. Both oxidants cause the synthesis of hemichrome, heme breakdown, and the release of free iron, which acts as a catalyst for processes involving ROS generation [38]. In conclusion, ROS signaling pathways in oil–erythrocyte interactions play a vital role in retaining their health status. Nevertheless, our results indicated that, in the case of solvent extraction, the oxidative stress of the cells (at least, as this can be described by ROS) is not enough to explain the hemocompatibility.

### 3.4. Inhibitory Effect of Mullet Roe Oils towards TRAP-6-Induced Aggregation in Human Platelet-Rich Plasma (hPRP)

In vitro analyses established that all mullet roe oil extracts exhibited strong inhibition against the TRAP-6 pathway of platelet aggregation in hPRP at very low doses (Table 3). Table 3 depicts the IC_50_ values (µg) of each sample in response to platelet activation induced by TRAP-6 and these values reflect the inhibitory capacity of each mullet roe extract. IC_50_ is the concentration at which a substance—in this case, the oil extract—exerts half its biological effect. Lower IC_50_ values imply a stronger inhibition of platelet aggregation in human plasma in response to a platelet agonist (TRAP-6). When comparing the two aggregometer channels, a sharp decline in platelet aggregation was observed in the cuvette where PRP was preincubated with the oil extract versus the cuvette where maximum reversible aggregation was induced by TRAP-6. Thus, we observed a strong inhibitory effect in human platelets incubated with fish oils versus platelet activation induced by TRAP-6 alone. As can be seen in Table 3, although there was no statistically significant difference in the oils due to the extraction methods, the lowest IC_50_ appeared to be from the sample prepared using the expeller oil press extraction method followed by the solvent extraction method. Lower IC_50_ values indicated a higher potency of the oils with regards to antiplatelet activity as less of the sample was required to cause an inhibition of platelets. Thus, considerable inhibition of platelet activity by mullet roe extract was observed in human PRP. No statistically significant differences were observed in platelet activity between the tested samples, indicating that the extraction procedures did not significantly affect the biological activity of the oils. This was consistent with previous findings, where lipids derived from marine fish (salmon) displayed significant inhibition against platelet-activating factor (PAF) and thrombin pathways [39]. It also agreed with studies showing the anti-inflammatory properties of fish-roe extracts [40]. In this case, however, inhibition against platelet activation through the thrombin pathway was more potent compared with salmon and other fish lipids [39,41].

### 3.5. Blood Clotting Time (BCT)

**Figure 5:**
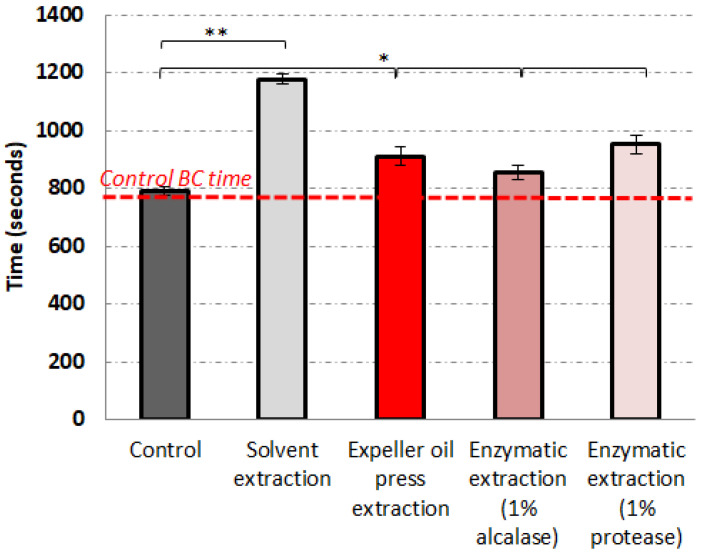
Whole blood clotting time after incubation with the tested oils. Bars indicate the standard deviation of each measurement (*n* = 3). Statistically significant differences between the control (without treatment) and the different tested oils are shown with brackets. * = *p* < 0.05; ** = *p* < 0.01.

Figure 5 presents the BCT of whole blood with and without contact with the oils extracted using different methods. The BCT of whole blood without the presence of the tested oils was found to be 790 s, which was in agreement with the literature [27]. The BCTs of the oils obtained using solvent extraction, expeller oil extraction, and enzyme-assisted extraction using protease and alcalase were 1180 ± 15 s, 913 ± 34 s, 855 ± 25 s, and 954 ± 32 s, respectively. The difference between the clotting time in the presence of all examined oils was statistically significant according to the *t*-test at α = 0.05. A statistically significant (*p* < 0.05) increase in the ΒCT in the presence of the solvent extract in comparison with the control and the other tested oil extracts was observed, followed by expeller oil press extraction and enzyme-assisted extraction using protease. Enzyme-assisted extraction using alcalase presented the lowest blood clotting time in comparison with the control. The blood clotting results were in agreement with our prior results and the strong inhibition against the TRAP-6 pathway of platelet activation in human plasma. These results indicated that, in vitro, all the extracted oils could inhibit the formation of platelet aggregates.

## 4. Conclusions

To the best of the authors’ knowledge, this study is the first to test the erythrocyte viability of oils from striped mullet roe byproducts. The impact of the extraction method on hemocompatibility was studied using four distinct extraction techniques. The best hemocompatibility without erythrocyte membrane changes was demonstrated by the expeller press oil extract and alcalase-assisted extraction. In samples where hemolysis was observed, changes in the membrane architecture were observed as well. Hemolysis could not be attributed solely to oxidative stress; at least, the latter could be depicted via the ROS. Regarding the relation of hemocompatibility with oil oxidation, there was no direct relationship between oil oxidation and hemocompatibility, but different oil production methods produced oils with different degrees of hemocompatibility. The green process of expeller press oil production provided the best results for hemocompatibility. Platelet aggregation assays demonstrated strong inhibition against the TRAP-6 pathway of platelet activation in human plasma for all four oils extracted using the different extraction techniques. The tested oil samples displayed an increased blood clotting time due to the observed inhibition of platelet aggregation. This is beneficial for their use in the development of sustainable cardioprotective nutraceuticals. However, further studies, including clinical investigations, are required to establish the health benefits of such oil extracts sourced from fish byproducts.

## Figures and Tables

**Table 1:** Extraction methods and conditions.

Mullet Roe Oil		
Extraction Method	Conditions	References
Expeller oil press extraction (EP)	T = 50 °C; centrifuging at 4132× *g* for 15 min	[8,9]
Solvent extraction (SE-25)	T = 25 °C for 2 h two times with ethanol; evaporation at 37 °C and 5 mbar	[8,9]
Enzymatic extraction (alcalase) (EE)	300 g; enzyme inactivation at 90 °C for 10 min with Alcalase^®^ 1%; incubation for 35 min at 55 °C; enzyme inactivation for 10 min at 90 °C; cooling at 25 °C; centrifuging at 8000 rpm for 15 min at 25 °C	[14]
Enzymatic extraction (protease) (EE)	300 g; enzyme inactivation at 90 °C for 10 min with Protease^®^ 1%; incubation for 35 min at 55 °C; enzyme inactivation for 10 min at 90 °C; cooling at 25 °C; centrifuging at 8000 rpm for 15 min at 25 °C	[14]

**Table 2:** Oxidation indices of the oil extracts.

Oils	PV (meq_O2_/Kg)	K_232_ (-)	K_268_ (-)	AV (-)	TOTOX (-)
Expeller oil press extraction	2.4 ± 0.0 ^a^	2.95 ± 0.00 ^a^	0.24 ± 0.00 ^a^	6.3 ± 0.0 ^a^	11.1 ± 0.0 ^a^
Solvent extraction	2.9 ± 0.0 ^b^	3.38 ± 0.00 ^b^	0.39 ± 0.00 ^b^	8.7 ± 0.1 ^b^	14.0 ± 0.1 ^b^
Enzyme-assisted extraction (1% alcalase)	2.5 ± 0.7 ^c^	3.00 ± 0.02 ^c^	0.47 ± 0.00 ^c^	16.9 ± 0.1 ^c^	21.9 ± 1.5 ^c^
Enzyme-assisted extraction (1% protease)	3.5 ± 0.7 ^d^	4.70 ± 0.01 ^d^	0.49 ± 0.02 ^d^	17.8 ± 0.9 ^d^	24.5 ± 0.5 ^d^

PV: peroxide value; AV: anisidine value; TOTOX: total oxidation value. All measured parameters presented statistically significant differences according to the analysis of variance (*p* < 0.05). ^a–d^ indicate differences according to Tukey’s test.

**Table 3:** The in vitro bioactivity of freshly extracted mullet roe oil extracted using various techniques against thrombin receptor-activating factor (TRAP-6)-induced human platelet aggregation, expressed as an IC_50_ in micrograms (µg) of the oil extract. All experimental analyses were carried out in triplicate (mean ± SD; *n* = 3). Differences were not statistically significant, according to the analysis of variance.

Oils	IC_50_ (µg) ± SD against TRAP-6-Induced Platelet Aggregation
Solvent extraction	0.31 ± 0.18
Expeller oil press extraction	0.3 ± 0.15
Enzymatic extraction (1% alcalase)	0.56 ± 0.42
Enzymatic extraction (1% protease)	0.41 ± 0.31

## Data Availability

The data presented in this study are available on request from the corresponding author. The data are not publicly available due to their complex nature.
